# Exploring Resistance to ETS Targeting Agents in Diffuse Large B‐Cell Lymphoma

**DOI:** 10.1002/cam4.71935

**Published:** 2026-05-13

**Authors:** Filippo Spriano, Luciano Cascione, Chiara Tarantelli, Giulio Sartori, Alberto J. Arribas, Adriana Velasova, Sara Napoli, Ondrej Havranek, Jeffrey A. Toretsky, Francesco Bertoni

**Affiliations:** ^1^ Institute of Oncology Research, Faculty of Biomedical Sciences USI Bellinzona Switzerland; ^2^ SIB Swiss Institute of Bioinformatics Lausanne Switzerland; ^3^ Biocev, First Faculty of Medicine, Charles University Prague Czech Republic; ^4^ First Department of Medicine ‐ Hematology, First Faculty of Medicine Charles University and General University Hospital Prague Czech Republic; ^5^ Departments of Oncology and Pediatrics Lombardi Comprehensive Cancer Center, Georgetown University Washington DC USA; ^6^ Oncology Institute of Southern Switzerland, Ente Ospedaliero Cantonale Bellinzona Switzerland

**Keywords:** diffuse large B‐cell lymphoma, ETS, MDR, resistance, venetoclax, XPO1

## Abstract

Diffuse large B‐cell lymphoma (DLBCL) remains a challenging disease with limited therapeutic options beyond standard immunochemotherapy. ETS transcription factors, including SPIB and SPI1, are implicated in lymphoma pathogenesis and can be targeted by the small molecule TK216, which disrupts ETS–DHX9 interactions. To explore mechanisms of resistance, we generated stable TK216–resistant clones from the ABC‐DLBCL line U2932. Resistant clones exhibited a 4–5‐fold increase in IC_50_ values and lost the ability to undergo G2–M arrest upon treatment. Transcriptomic and mutational analyses revealed three resistance patterns: (i) MDR1/ABCB1 overexpression, leading to multidrug efflux; (ii) Cluster A, enriched for proliferation, Wnt, and transcriptional programs, with mutations in ESR2, USP24, and SFSWAP; and (iii) Cluster B, characterized by actin/microtubule remodeling, altered metabolism, and mutations in SRSF11 and PATJ. Pharmacologic screening revealed an increased sensitivity of resistant cells to BCL2, MCL1, and XPO1 inhibitors, while also showing reduced sensitivity to aurora kinase and microtubule‐targeting agents. Venetoclax and selinexor retained activity in resistant models, supporting their potential for rational combinations with TK216. These findings demonstrate that multiple, heterogeneous mechanisms drive resistance to ETS inhibition in DLBCL, highlighting therapeutic strategies to overcome it.

## Graphical abstract

This study provides the first systematic characterization of resistance to ETS inhibition in diffuse large B‐cell lymphoma (DLBCL). Using TK216‐resistant models, we reveal heterogeneous escape mechanisms, including MDR1 upregulation, transcriptional reprogramming, and cytoskeletal remodeling, each associated with distinct mutational signatures. Importantly, pharmacologic profiling identified vulnerabilities to BCL2, MCL1, and XPO1 inhibitors, highlighting rational strategies to overcome resistance. These findings extend the translational potential of ETS targeting in lymphoma and establish a framework for biomarker‐guided combination therapies in future clinical development.

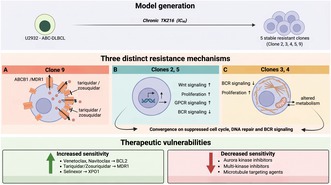

## 1 Introduction

Diffuse large B‐cell lymphoma (DLBCL) is the most common lymphoma subtype, accounting for approximately 30%–35% of all cases [1]. Despite significant advances in the management of DLBCL patients [2, 3, 4], there remains a need for therapies with novel mechanisms of action. Targeting ETS (E26 transformation‐specific) factors is supported by the notion that this family of transcription factors plays a crucial role in regulating cell growth, differentiation, and survival across various cell types and tissues [5]. In DLBCL, dysregulation of ETS1, SPIB, and FLI1, members of the ETS transcription factor family, has been linked to the pathogenesis of DLBCL [6, 7, 8, 9, 10, 11, 12]. Based on YK‐4‐279, TK216 is a first‐generation clinical‐grade small molecule that targets the EWS‐FLI1 fusion protein [13, 14]. TK216 demonstrated clinical activity in Ewing sarcoma patients as a single agent or in combination with vincristine; however, further optimization is required, including the development of an oral formulation [15]. We have previously reported the potent in vitro and in vivo anti‐tumor activity of TK216 in DLBCL models [13]. In DLBCL cells, TK216 exerts its activity by disrupting the interaction between the ETS factors SPIB or SPI1 and the RNA helicase DHX9 in activated B‐cell‐like (ABC) or germinal center B‐cell‐like (GCB)‐DLBCL, respectively [13]. To better understand the mechanism of action of TK216 in DLBCL cells and identify ways to improve its efficacy, we have developed models of primary resistance to the compound.

## 2 Materials and Methods

### 2.1 Cell Lines and Drug Treatments

All cell lines used in this project were cultured under standard conditions (37°C and 5% CO_2_ in a humidified atmosphere). Cell lines were obtained and maintained in the appropriate medium supplemented with 10% Fetal Bovine Serum (FBS) (FBS‐11A, Capricorn Scientific, Germany) and 1% penicillin/streptomycin (15,070,063, Thermo Fisher Scientific, Switzerland). All cell lines were periodically tested to confirm Mycoplasma negativity using the MycoAlert Mycoplasma Detection Kit (Lonza, Visp, Switzerland). Additionally, all cell lines were validated for identity using short tandem repeat (STR) DNA fingerprinting at IDEXX BioResearch (Ludwigsburg, Germany) or with the Promega GenePrint 10 System kit (B9510).

The in vitro anti‐proliferative activity was determined by treating the cells for 72 h. After 3 days, the MTT solution was added to the plates. After 4 h, the reaction was stopped with SDS (25% solution), and absorbance was measured with Cytation 3 (Agilent, United States). IC50 calculations were performed using an in‐house R script.

Live‐cell imaging treatments were performed using an Incucyte machine (Sartorius). 30,000 cells per well were seeded in a 96‐well plate and treated with TK216 (350 nM), Tariquidar (5 μM), or Zosuquidar (5 μM) at single or combined doses and monitored for approximately 4 days. TK216, YK‐4‐279, and Tariquidar were purchased from MedChemExpress; zosuquidar from LubioBioscience; and all other compounds from Selleckchem.

### 2.2 Resistance Induction

To induce resistance to TK216, an ABC‐DLBCL cell line, U2932, was seeded in a 24‐well plate at 50,000 cells/mL per well and treated with an IC90 concentration (500 nM) of TK216. In parallel, as a control, three flasks of U2932 were treated with the same volume of DMSO. After the cells recovered from the treatment, they were treated periodically for almost a year until clones with stable TK216 resistance developed. We assessed the identity of the cell lines to determine whether contamination with other cells had occurred during the continuous passages. Resistant and parental cell lines were tested by short tandem repeat DNA fingerprinting using the Promega GenePrint 10 System kit (B9510), showing that all clones derived from the parental U2932 cell line.

### 2.3 Combination Studies

All combinations were tested by treating cells for 72 h with increasing concentrations of both compounds in a 7 × 7 matrix, with three technical replicates, followed by an MTT assay and SDS cell lysis. The combination index (CI) was calculated using the Chou‐Talalay [16], HSA, ZIP, Loewe, and Bliss methods [17].

### 2.4 Cell Cycle

After the desired treatment time, cells were harvested and washed twice with PBS. The cell pellet was collected, resuspended in 1 mL of PBS, and fixed with 4 mL of molecular‐grade 100% ethanol (CAS: 64–17‐5, VWR, Belgium). The ethanol was added dropwise to the cells under constant, gentle agitation. Cells were then incubated for at least one night at −20°C. Subsequently, cells were washed with PBS +1% FBS and incubated with propidium iodide for at least 10 min in the dark at 37°C. Data acquisition was performed using the BD FACSCanto flow cytometry system (BD Bioscience), and data were analyzed with FlowJo software.

### 2.5 RNA Extraction

Total RNA was extracted using TRIzol reagent (Invitrogen) according to the manufacturer's protocol. During RNA isolation, samples were treated with RNase‐free DNase (Qiagen, Hilden, Germany) to remove potential genomic DNA contamination. RNA concentration was measured using a Nanodrop spectrometer (DS‐11 spectrophotometer, DeNovix) at 260 nm.

### 2.6 Transcriptome Profiling and Data Mining of RNA‐Seq for Identification of Resistance‐Associated Mutations

RNA‐seq read quality was assessed using FastQC (v0.11.5), and low‐quality reads and bases, along with adapter sequences, were removed using Trimmomatic (v0.35). The trimmed, high‐quality reads were aligned using STAR [18], a spliced‐read aligner that allows reads to span multiple exons. On average, we aligned 85% of the reads for each sample to the reference genome (HG38). The HTSeq‐count software package [19] was then used for gene‐level expression quantification. Differential expression analysis was performed on gene‐level read count data using the ‘limma’ pipeline [20]. We first subsetted the data to genes with counts‐per‐million values greater than one in 3 or more samples. The data were normalized per sample using the ‘TMM’ method from the edgeR package [21], and then transformed to log2 counts‐per‐million using the edgeR function ‘cpm’. Linear model analyses, with empirical‐Bayes moderated estimates of standard error, were used to identify genes most associated with the phenotype of interest, and an FDR‐adjusted *p*‐value of < 0.05 was set as the threshold for statistical significance.

Reads in fastq format were preprocessed with GATK version 3.5 to remove Illumina adapter sequences (analysis type –T ClipReads, −XF illumina.adapters.fa) and to filter out Phred‐scaled base qualities of 10 or lower (−QT 10), as previously described [22]. After GATK processing, reads were mapped to HG38 using STAR (basic 2‐pass method). SAMtools [23] flagstat was used to compute the number and percentage of reads mapped to the genome. PCR/optical duplicates were marked with Picard (http://broadinstitute.github.io/picard/). Base quality recalibration and indel realignment were performed using GATK [24]. Variant calling was performed using MuTect version 1.1.4 [25] for parental/resistant pairs. Variants (single‐nucleotide mutations and indels) were annotated using Annovar [26]. Variant filtering was performed using the following criteria: exonic, nonsynonymous, somatic, and damaging variants, excluding variants found in repetitive regions, regions with poor coverage, and reported SNPs.

### 2.7 Gene Set Enrichment Analysis (GSEA)

Log2‐transformed and normalized expression profiles were used in Gene Set Enrichment Analysis (GSEA) [27] to functionally annotate differentially expressed genes. Standard settings were used for either regular GSEA or preranked GSEA (when using a ranked list organized by fold change), and signatures with nominal *p*‐values < 0.05 and FDR < 0.05 were considered statistically significant. The ssGSEA data from Gene Set Enrichment Analysis (GSEA) [27] were used as input for the Cytoscape software. Heatmaps were generated using the R pheatmap package.

### 2.8 Multidimensional Scaling (MDS) Plot and Heatmap

Analyses were performed using the R environment (RStudio console; RStudio, Boston, MA). Pearson's clustering method was used for heatmaps.

## 3 Results

### 3.1 The ABC‐DLBCL Cell Line U2932 Develops Resistance to TK216 in Multiple Ways

**FIGURE 1:**
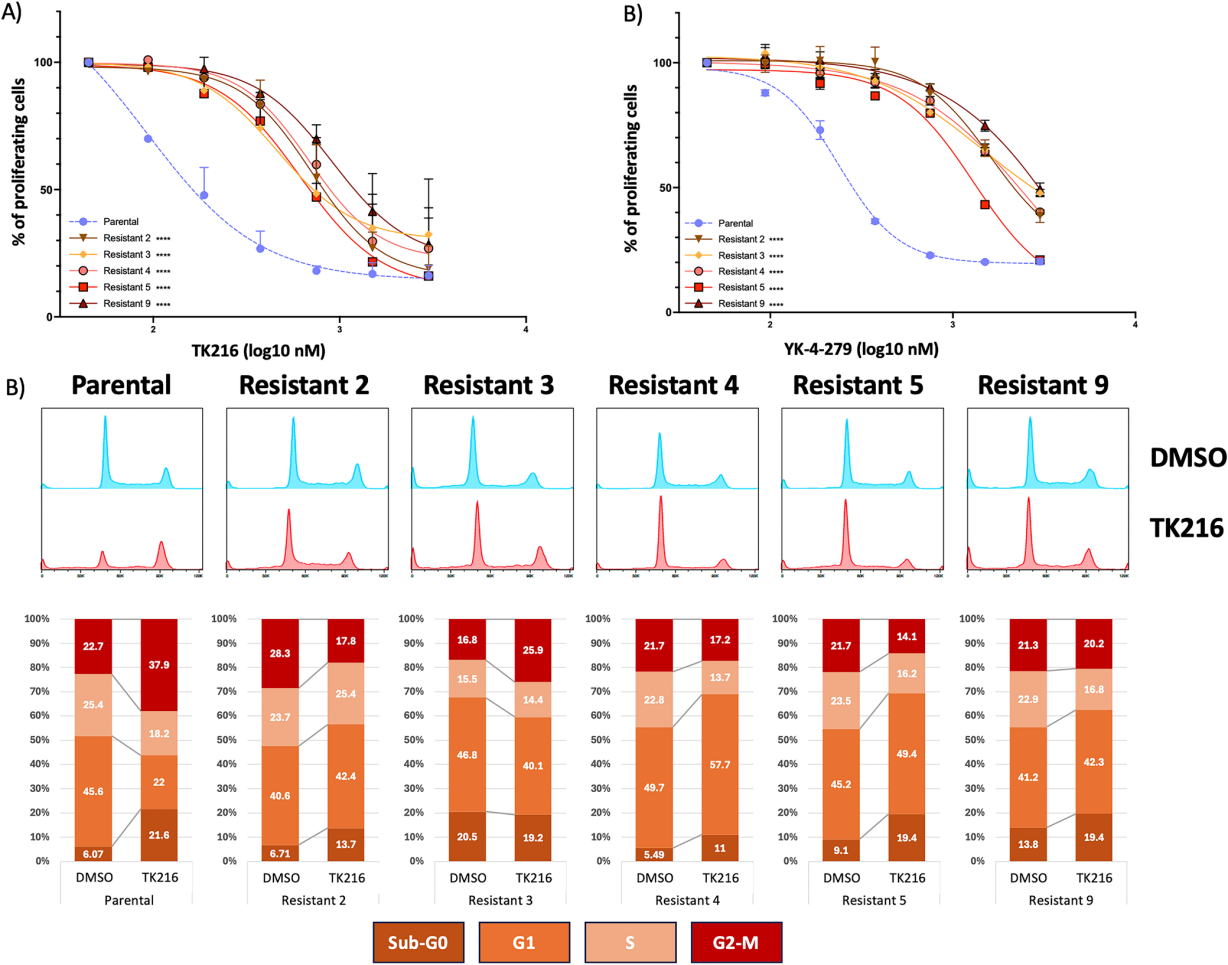
U2932 developed resistance to TK216. Resistant clones were treated with increasing doses of TK216 (A) or YK‐4‐279 (B) for 72 h after a 2‐week washout from the small molecule to validate the obtained resistance. Statistical analysis was performed using an extra‐sum‐of‐squares F test comparing parental and resistant cells. Experiments performed in at least two replicates. (C) Cell cycle performed in parental cell line compared to resistant clones after 24 h of treatment with TK216, 500 nM, or DMSO. The figure shows representative results from experiments performed in duplicate.

To induce resistance to TK216, one ABC‐DLBCL (U2932) and one GCB‐DLBCL cell line (DOHH2) were seeded at time 0 into 24‐well plates and treated with TK216 at 500 nM and 1.5 μM, corresponding to the respective IC90 concentrations determined by 72 h drug treatment. After almost 2 months, only cells from 5 of 24 wells in U2932 were recovered following the initial TK216 treatment. None of the 24 DOHH2 replicates developed stable resistance to TK216. The U2932‐resistant cells (defined as clones 2, 3, 4, 5, and 9) were then passaged and treated again with TK216 (500 nM) every 3 days for at least 6 months. The resistance was stable, as demonstrated by the MTT assay performed on cells maintained in drug‐free medium for at least 2 weeks. The cells exhibited a 4‐ to 5‐fold increase in their IC50 values compared with their parental counterparts (Figure 1A). The average IC50 change was from 150 nM to 1 μM. In addition, cells became resistant to YK‐4‐279 (Figure 1B), the ETS inhibitor from which TK216 is derived [28], but not to a drug with a completely unrelated mode of action, such as vorinostat (Supplementary Figure S1).

The acquisition of resistance was also demonstrated by performing a cell cycle analysis after 24 h of TK216 treatment at 500 nM (IC90 of the parental cell lines) (Figure 1C). The cell cycle analyses revealed that resistant clones did not undergo G2‐M cell cycle arrest but maintained only a minimal sub‐G0 peak, indicating a smaller subset of cell death than in parental cells.

**FIGURE 2:**
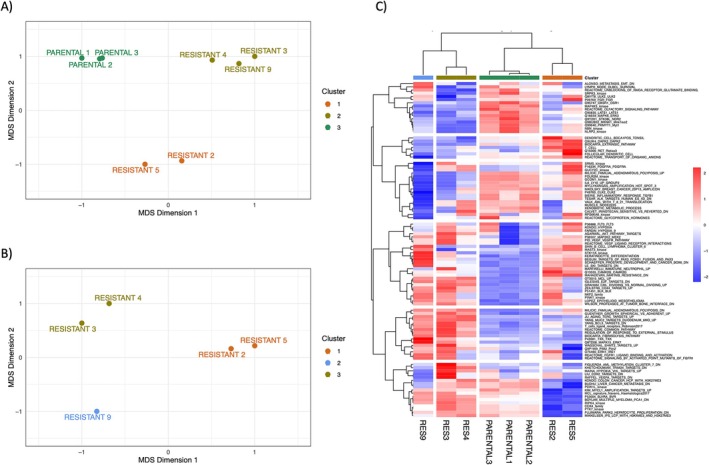
Multidimensional scaling (MDS) plot performed on RNA‐Seq data on parental cell lines and resistant clones (A) and corresponding unsupervised clustering of ssGSEA (B). MDS plot based on RNA‐seq data performed on (A) parental and resistant clones and (B) only in resistant clones. (C) Unsupervised clustering of single‐sample gene‐sets enrichment data in parental and resistant cell lines. Pearson's clustering method was used; the top 100 gene sets with the highest standard deviation are plotted.

The five resistant clones and three biological replicates of the parental cell line underwent RNA‐Seq to identify transcriptomic changes associated with decreased sensitivity to TK216 (Table S1). RNA extraction was performed in the absence of TK216 treatment for at least 2 weeks to capture stable differences. The MDS (multidimensional scaling) plot identified three main groups of cells: one comprising the three parental cell lines, another comprising Clone 2 and Clone 5, and a third comprising Clone 3, Clone 4, and Clone 9 (Figure 2A). Focusing only on resistant cells, these were divided into three clusters: Clone 2 and Clone 5 (Cluster A), Clone 3 and Clone 4 (Cluster B), and Clone 9 (Figure 2B). We then performed an ssGSEA analysis of parental and resistant cell lines, followed by unsupervised clustering (Figure 2C). The clustering confirmed the MDS data, highlighting four clusters: parental, clones 2–5 (Cluster A), 3–4 (Cluster B), and clone 9.

The detection of three clusters in resistant clones suggests that resistance to TK216 inhibition can be achieved through alternative adaptive states that may require distinct therapeutic approaches.

### 3.2 Resistance to TK216 Can Be due to Multidrug Resistance Efflux Pumps

**FIGURE 3:**
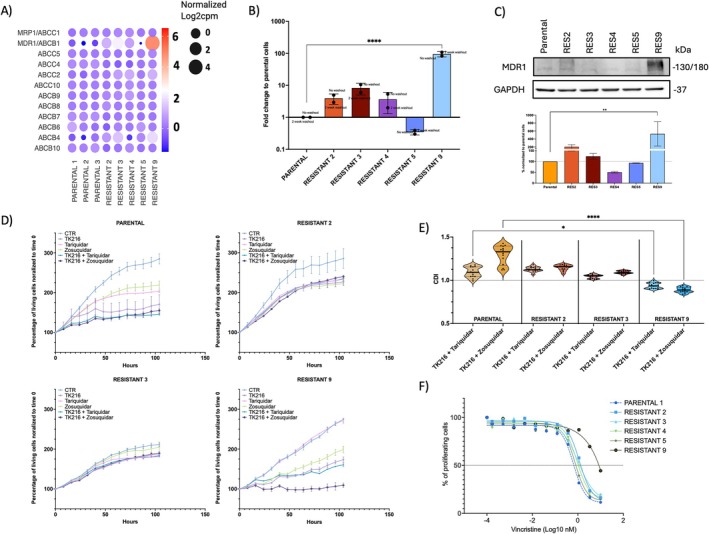
TK216 can serve as a substrate of multidrug resistance efflux pumps. (A) Median normalized Log2 expression of genes coding for proteins involved in multidrug resistance. (B) MDR1 expression was obtained with real‐time PCR and performed on resistant clones under TK216 or after 2 weeks of washout. *Y*‐axis, Fold change between parental cell lines and resistant clones. The Krustal‐Wallis test was performed. (C) Protein MDR1 expression with the respective quantification. Representative of two biological replicates. (D) Representative growth curve of cells treated with TK216 or MDR1 inhibitors (tariquidar and zosuriquidar) in single or combination. On these curves, we calculated the (E) Coefficient of drug interaction (CDI), CDI < 1 = synergism, CDI = 1 = additive, CDI > 1 = antagonism. CDI was calculated on two independent experiments. The Krustal–Wallis test was performed. (F) Dose–response curve obtained after 72 h of treatment with vincristine in parental and resistant clones. The figure shows representative results from duplicate experiments.

To identify the mechanisms underlying resistance, we first evaluated the expression of MDR genes that encode proteins involved in drug efflux. Based on RNA‐Seq data, we identified MDR1 (ABCB1) as being expressed much more in clone 9 than in the parental cell lines and the remaining resistant clones (Figure 3A). MDR1 was also upregulated in clones 2, 3, and 4, but at a lower level than in clone 9. The RNA‐Seq data were further validated by real‐time PCR, which showed higher expression, normalized to parental levels, of MDR1, especially in clone 9. The upregulation was present in resistant clones under TK216 treatment selection and in clones with more than 2 weeks of drug washout, demonstrating that this is a stable mechanism acquired during the time required to survive TK216 administration (Figure 3B), and not a transient effect induced by TK216 exposure. The RNA data were further validated by immunoblot, showing a 2‐ and 1.5‐fold upregulation of MDR1 protein in clones 2 and 3, respectively, compared to parental cells, and a 6‐fold increase in clone 9 (Figure 3C). Using live‐cell imaging, treatment of resistant cell lines (resistant 2, 3, and 9) with MDR1 inhibitors tariquidar and zosuquidar showed that clone 9 benefited from combination treatments compared to single‐drug treatments (Figure 3D). This was also confirmed by a coefficient of drug interaction (CDI) < 1 (Figure 3E). Finally, treatment with vincristine, a well‐known substrate of various MDR proteins and especially of MDR1 [29, 30], showed a significant increase in resistance in TK216‐resistant clone 9, yet much smaller decreases in sensitivity in the remaining resistant clones (Figure 3F). These experiments further demonstrate that resistance in clone 9 was driven by overexpression of the efflux pump MDR1.

### 3.3 Characterization of Cluster A and Cluster B Resistant Clones

Clones 2 and 5 are analyzed together as Cluster A, and clones 3 and 4 are interpreted as Cluster B. In Cluster A, the top 50 genes whose expression was reduced compared to parental cells included genes involved in membrane organization and cell morphology (FMNL2, ACTN1, DBN1, and ADD2); zinc finger proteins (ZNF57, ZNF470, ZBTB32, and ZNF792); and VASH1 (involved in microtubule dynamics). The top 50 genes whose expression was increased in resistant cells included genes involved in cell proliferation (SAMSN1, PITX2, MEOX2, and MECOM); genes involved in calcium binding or channel, such as SPOCK2, CNGB3, EFCAB12, and TRPC4; genes involved in the WNT pathway (TPB, LGR6, and CXXC4); genes involved in transcriptional regulation (TFEC, ZFP30, ZNF154, TWIST1, ID4, FOXP2, and MECOM); and genes involved in microtubule formation (MAP2, KIF13A, and MAP7) (Figure S2A).

In Cluster B, the top 50 genes whose expression was reduced compared to parental cells included genes involved in microtubule dynamics (FSD1, CAMSAP2, CENPE, MID1IP1, GPHN); zinc finger proteins (ZNF354B, ZNF234, ZNF57), and the actin‐related gene ACTN1. We identified actin‐binding genes among the top 50 upregulated genes, such as SYNPO, DMTN, and FSCN2 (Figure S2B).

Supplementary Figure 3 summarizes the gene sets enriched among transcripts differentially expressed between resistant and parental cells. Transcripts upregulated in Cluster A were enriched in the hypoxia pathway, extracellular matrix‐associated genes, the interferon alpha pathway, secreted factors, genes upregulated after treatment with PI3K or BET inhibitors, GPCRs, genes upregulated after IRF4 knockdown, genes repressed by SPIB, and genes modulated after lenalidomide treatment. We also observed downregulation in pathways such as DNA repair, cell cycle and mitosis, Aurora kinase A activation, MYC targets, genes involved in the spliceosome and ribosome machinery, and genes involved in the B‐cell receptor (BCR) pathway.

In Cluster B, upregulated transcripts, similar to those observed in Cluster A, were enriched for extracellular matrix‐associated genes, genes modulated following lenalidomide treatment, genes upregulated after treatment with PI3K or BET inhibitors, and GPCRs. In contrast to Cluster A, genes were downregulated after IRF4 or SPIB knockdown, and metabolic pathways were upregulated. Among the downregulated targets, we identified genes downstream of BCR, as well as genes downregulated after PI3K inhibitor treatment, including those involved in the cell cycle, mitosis, DNA repair, and aurora kinase A.

The downregulation of BCR‐related genes and upregulation of GPCRs was confirmed by an increase in p‐AKT at the protein level and a decrease in p‐BTK (Figure S4).

To identify differences or similarities in pathways modulated between Cluster A and Cluster B, we performed an enrichment map analysis using Cytoscape software (Figure S5).

Although Cluster A and Cluster B resistant clones displayed distinct transcriptional signatures, pathway‐level analyses revealed convergence on common biological processes, including suppression of cell cycle and DNA repair pathways and rewiring of survival signaling. This suggests that distinct upstream regulatory events may ultimately funnel into a limited number of adaptive states compatible with survival under ETS inhibition.

Because we identified pathways associated with secreted factors among the gene sets upregulated in both clusters, and because we previously documented that secreted factors can drive lymphoma drug resistance [31, 32], we treated parental cell lines with TK216 in the presence of conditioned media from resistant cell lines. No effect was observed (Figure S6).

Using the RNA‐seq data, we also analyzed the mutational status of parental and resistant cells and confirmed the cluster groupings observed by gene expression (Figure S7A). Focusing only on missense mutations with protein‐coding implications, we identified ESR2, USP24, LRCH1, FAM200A, SFSWAP, and TRAPPC11 as commonly mutated in resistant clones 2 and 5. In contrast, FCGR2B, SRSF11, and PATJ were mutated in resistant clones 3 and 4 (Figure S7B).

### 3.4 Pharmacological Screening of Resistant Clones

To identify compounds with differential sensitivity between resistant and parental cells, we performed pharmacological screening using a library of 348 compounds in resistant clone 2, representative of Cluster A, and resistant clone 3, representative of Cluster B, compared with a single parental cell line. As previously reported [33], the library included approved kinase inhibitors, epigenetic compounds, and small molecules targeting critical biological pathways, such as the PI3K/AKT/MAPK signaling pathway and apoptosis (Table S2). Cells were treated with two concentrations of each library compound (500 nM and 50 nM) for 72 h. Compounds associated with a 15% or greater difference in cell proliferation between parental and resistant cell lines were selected. Despite belonging to two different clusters, the two resistant clones exhibited similar behavior, in agreement with the similarities observed in deregulated pathways.

**FIGURE 4:**
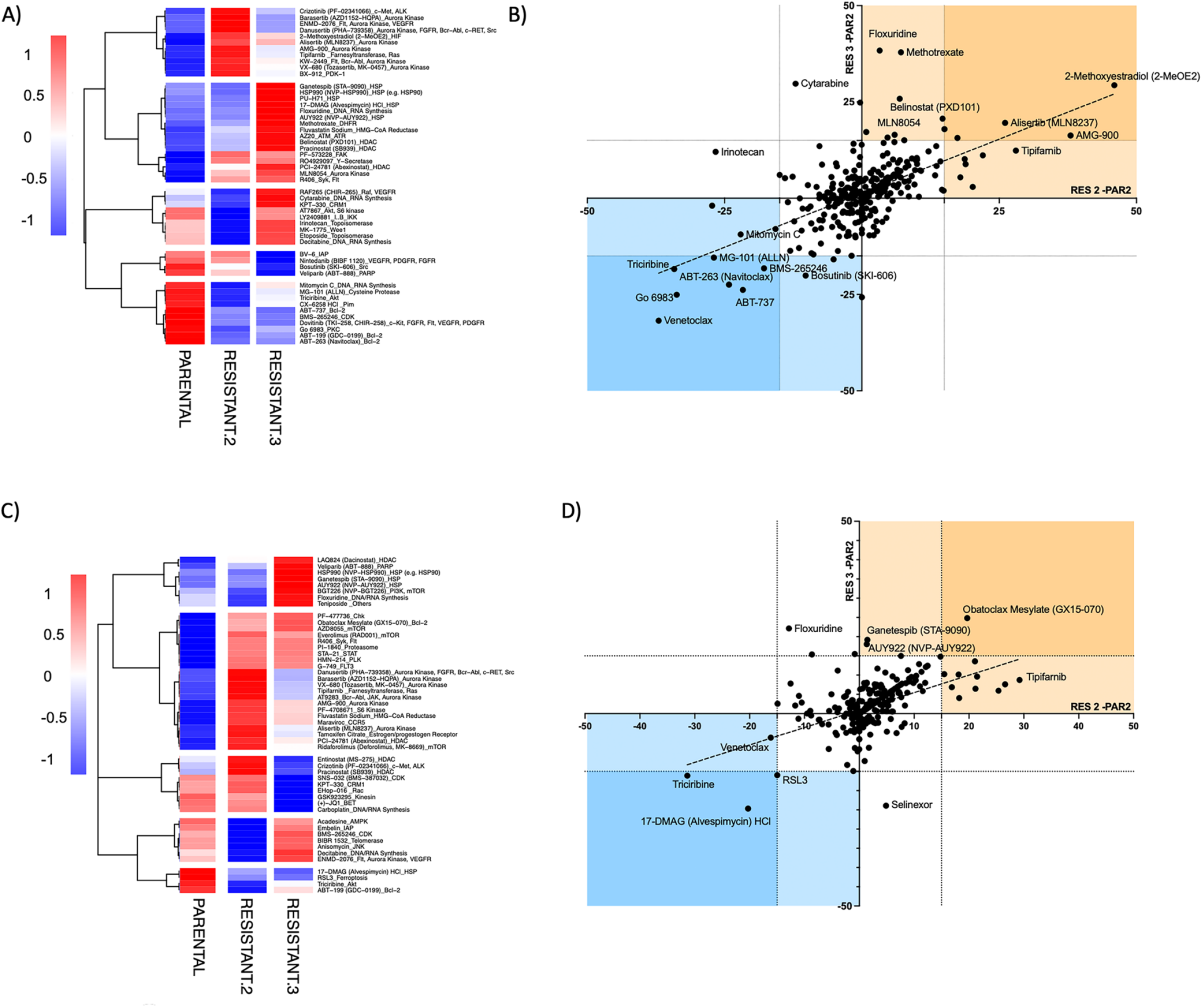
Pharmacological screening in TK216‐resistant clones. Pharmacological screening was performed with library compounds 500 nM (A, B) or 50 nM (C, D). In the heatmaps (A, C), the top 50 drugs with higher standard deviations are shown.

Compounds with greater activity in resistant clone 2 than in parental cells included inhibitors of BCL2, PI3K/AKT, topoisomerase, PIM, and heat shock protein (HSP). Among the compounds showing reduced activity in the resistant clone, there were inhibitors of aurora kinase, HIF, HDAC, VEGFR, multikinase inhibitors, and 2‐Methoxyestradiol (Figure 4A,B, Table S2).

Clone 3 showed increased sensitivity to inhibitors of BCL2, AKT, VEGFR, IAP, HSP, and XPO1. In contrast, among the compounds showing reduced activity in the resistant clone, we identified inhibitors of Aurora Kinase or multikinase inhibitors, as well as inhibitors of DNA/RNA synthesis, HSP, HDAC, and ATM/ATR (Figure 4C,D, Table S2).

Finally, we identified anti‐metabolites (antifolates and pyrimidine analogs) and compounds targeting aurora kinase as less active in resistant cells, consistent with downregulation of mitosis and cell cycle genes, as well as aurora kinase A‐related pathways (Table S1).

**FIGURE 5:**
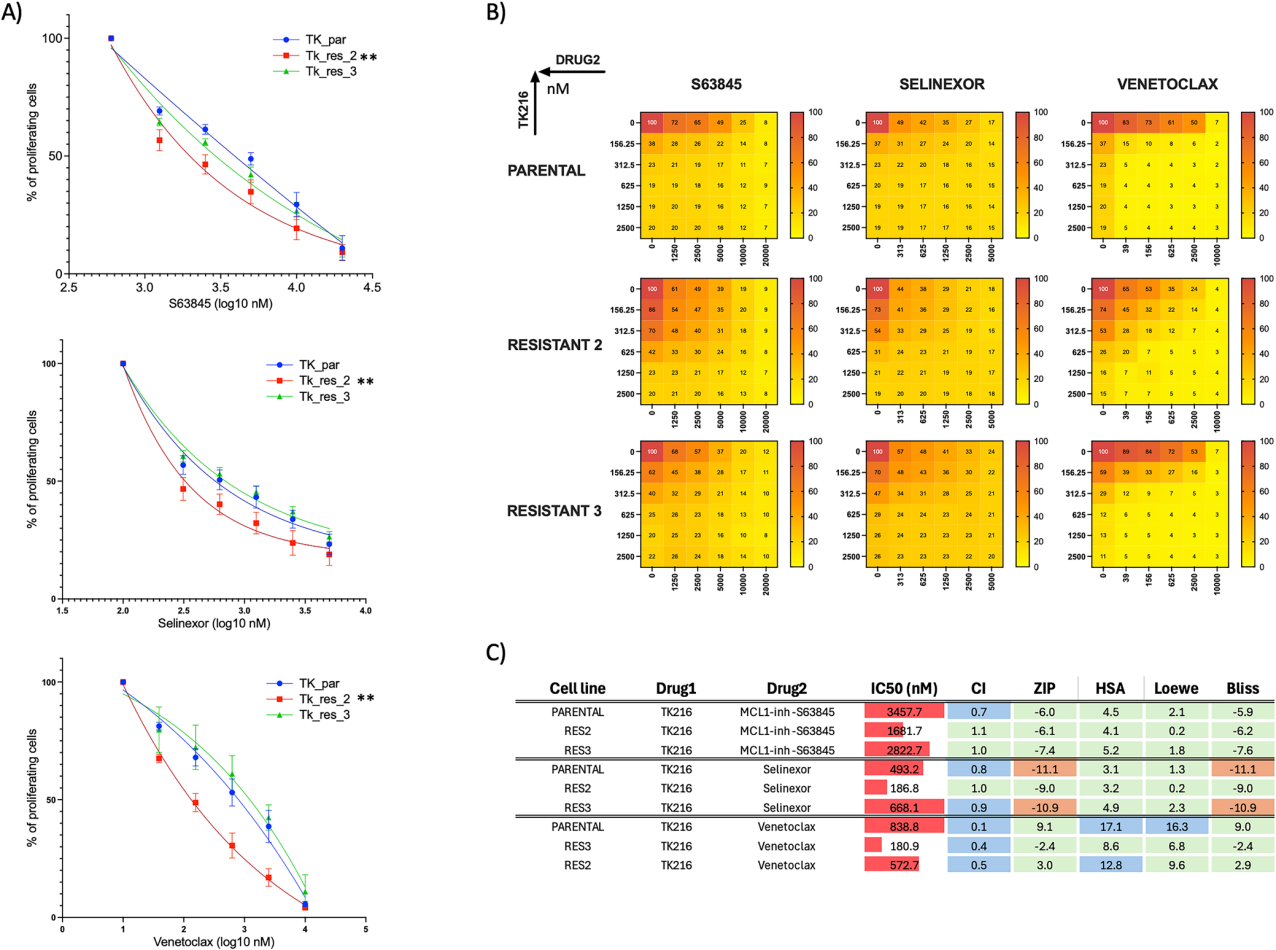
Single and combined treatment of venetoclax, S63845, and Selinexor in resistant clones 2 and 3 compared to parental 1. (A) Dose–response curve of venetoclax, S63845, and selinexor in resistant 2/3 and parental cell lines. Statistical analysis was performed using an extra‐sum‐of‐squares *F* test comparing parental and resistant cells. Experiment performed in at least two biological replicates. (B) Heatmaps showing the % of proliferating cells in combination experiments. (C) Table summarizing IC50s and combination values. Synergy: CI < 0.9; Additivity: 0.9 < CI < 1.1; No benefit/antagonism: CI > 1.1. Synergy: ZIP/HAS/Loewe/Bliss > 10; Additivity: −10 < ZIP/HAS/Loewe/Bliss < 10; No benefit/antagonism: ZIP/HAS/Loewe/Bliss < −10.

The observation that BCL2‐targeting compounds were more active in resistant cells than in parental cells was consistent with prior evidence showing that venetoclax is one of the few compounds that synergize with TK216 in DLBCL cells [13]. We confirmed the higher activity of venetoclax in resistant clone 2 cells via a dose–response experiment (Figure 5A). The increased sensitivity in resistant clone 2 reflected a baseline status of antiapoptotic proteins similar to that of venetoclax‐treated cells [13], with decreased BCL2 and increased MCL1 (Supplementary Figure 8). Surprisingly, the pan‐BCL2 inhibitor obatoclax was less active in both resistant cells. Since MCL1 is a distinct target of obatoclax relative to other molecules that exhibit increased activity in resistant cells, we assessed the activity of the MCL1 inhibitor S63845 as a single agent in resistant and parental cells, as well as in combination with TK216 (Figure 5A). However, the MCL1 inhibitor S63845 showed significantly increased activity in resistant clone 2 and a mild, nonsignificant increase in clone 3. In addition, the combination of TK216 and S63845 showed an overall benefit in both parental and resistant cells (Figure 5B,C).

Based on screening, the CRM1/XPO1 inhibitor selinexor showed higher activity in resistant clone 2 but not in clone 3, as confirmed in a dose–response experiment (Figure 5A). In our previous work, this compound, FDA‐approved for relapsed/refractory DLBCL patients [34], had an additive effect in two of four DLBCL cell lines tested, which did not include U2932. Here, we tested selinexor as a single agent in resistant and parental cells and in combination with TK216 in the U2932 cell line (Figure 5). Although we observed increased sensitivity to selinexor in resistant clone 2 compared with parental cells as a single agent, we did not observe a similar difference in combination with TK216. Selinexor showed benefit or synergy when combined with TK216 in parental and resistant clones.

## 4 Discussion

ETS‐targeting agents have demonstrated preclinical antitumor activity in ETS‐driven tumors, including DLBCL. TK216 is a first‐generation ETS‐targeting small molecule that completed a Phase 1 trial in patients with Ewing sarcoma. To identify resistance mechanisms patients may encounter, we developed five resistant clones through prolonged TK216 treatment and explored established anticancer drugs that mitigate TK216 resistance. One cell line, U2932, showed a range of changes that could sustain resistance. The magnitude of resistance, ranging from 150 nM (sensitive) to 1 μM (resistant), was significant, as the drug levels in the Phase 1 clinical trial achieved serum concentrations above that range with limited toxicity [15]. However, a second cell line, DOHH2, did not establish stable resistance to TK216.

Starting with the ABC‐DLBCL cell line U2932, which is TK216‐sensitive [13], we observed resistance in five of 24 wells of cells exposed to the IC90 of the ETS‐targeting small molecule for almost 2 months. The cells were then maintained for 6 months under TK216, and the resistance remained stable after 2 weeks of washout, attributed to three distinct mechanisms. The identification of three distinct resistance patterns highlights the biological heterogeneity of adaptive responses to ETS inhibition. One modality, represented by the resistant clone 9, involved overexpression of the multidrug resistance efflux pump MDR1 (ABCB1). Overexpression was observed in RNA‐seq data and confirmed by real‐time PCR and immunoblotting. MDR1 is an ATP‐dependent efflux pump that confers resistance to many drugs by increasing efflux of drugs from cells. Resistant clone 9 exhibited increased resistance to TK216, YK‐4‐279, and vincristine, a well‐known MDR1 substrate [29]. In addition, pharmacological inhibition of MDR1 increased sensitivity to TK216 in the resistant clone 9. These data indicate that TK216 and YK‐4‐279 are substrates of MDR1, whose overexpression confers resistance. Future studies directly measuring intracellular drug accumulation or transport will be required to formally confirm MDR1‐mediated export of TK216.

In the remaining four clones, only two (2 and 3) showed a modest increase in MDR1 expression but did not show a substantial difference in vincristine sensitivity. This suggests that MDR1 did not mediate their resistance to TK216 and that cells could escape TK216 through other mechanisms.

The other resistant clones were divided into two clusters based on their transcriptome profiles and mutational landscapes: Cluster A (clones 2 and 5) and Cluster B (clones 3 and 4). The two clusters exhibited distinct sets of upregulated and downregulated genes. However, these converged on similar pathways, likely relevant to surviving exposure to TK216. In all these clones, we observed upregulation of genes regulated by BET inhibitors, as well as genes modulated by SPIB and IRF4, and downregulation of genes related to DNA repair, the cell cycle, and splicing. Such pathway convergence, despite transcriptional divergence, is a well‐described phenomenon in drug resistance and likely reflects selective pressure toward a limited set of viable survival programs. From a therapeutic perspective, this convergence suggests that targeting shared downstream vulnerabilities may be more effective than focusing on individual gene alterations.

Among the genes mutated in cluster A, we identified ESR2 (estrogen receptor), ubiquitin‐specific protease USP24, the negative regulator of CDC42 (LRCH1), and the alternative splicing factor (SFSWAP). In contrast, in cluster B, we identified the splicing factor SRSF11 and PTAJ, which localizes proteins to the cell membrane. These mutations must be validated for their potential contribution to resistance to TK216. This is particularly the case for genes such as SRSF11 and SFSWAP, given the role of alternative splicing and the participation of some ETS factors in splicing mechanisms [35].

Performing a high‐throughput drug screen with a library of 348 FDA‐approved compounds, we identified compounds whose activity changed in resistant cells compared with parental cells. Among the compounds more active in resistant cell lines, we identified BCL2 inhibitors, particularly in resistant clone 2, which served as a representative of cluster A. The difference in BCL2 inhibitor sensitivity correlated with variations in the baseline expression of BCL2 and MCL1. This finding was consistent with our previous work [13], in which the BCL2 inhibitor venetoclax was one of the few compounds that synergized with TK216. Resistant clone 2 also showed increased sensitivity to the CRM1/XPO1 inhibitor selinexor and the MCL1 inhibitor S63845. Resistant clone 3, representative of cluster B, showed increased sensitivity to the MCL1 inhibitor S63845 too. Despite this increased sensitivity, we did not observe enhanced synergism when these compounds were combined with TK216. Of interest, despite increased sensitivity to venetoclax (BCL2), navitoclax (BCL2 and BCL‐XL), and S63845 (MCL1), we observed reduced activity of obatoclax (BCL2, BCL‐XL, and MCL1), suggesting that the resistant cells likely underwent a reprogramming of apoptotic dependencies, becoming reliant on specific anti‐apoptotic proteins like BCL2 or MCL1 rather than a broad set of these proteins. In these reprogrammed cells, selective inhibition of the dominant anti‐apoptotic protein can efficiently release proapoptotic factors and trigger apoptosis, whereas broad inhibitors with lower target affinity, such as obatoclax, may fail to effectively disrupt the key survival interaction or may activate compensatory stress responses.

YK‐4‐279 synergizes with the chemotherapeutic drug vincristine in Ewing's sarcoma cells [36]. Prior work has linked the development of YK‐4‐279 resistance to changes in gene expression; however, the affected genes were not studied in detail, and they do not overlap with our findings [37]. Povedano and colleagues developed resistance to TK216 in an Ewing Sarcoma cell line previously engineered to be DNA mismatch repair deficient and identified microtubules as potential targets of TK216 [38]; importantly, their experiments did not evaluate the drug's effect on EWS‐FLI1. We did not identify mutations in the gene coding for α‐tubulin, as reported [38]. However, one of the compounds with the most reduced activity in our resistant DLBCL cells was 2‐Methoxyestradiol (2‐ME2), which can inhibit tubulin polymerization by interacting at the colchicine site [39], indicating that tubulin deregulation might still contribute to resistance to TK216.

In conclusion, we have shown that multiple mechanisms may sustain resistance to TK216 in DLBCL cells exposed to high doses of the compound. Possible mechanisms include overexpression of the multidrug resistance efflux pump MDR1 and activation of multiple anti‐apoptotic proteins. However, none of the 24 DOHH2 replicates developed stable resistance to TK216, suggesting that resistance may be cell‐line or lymphoma‐subtype‐specific. Fortunately, resistance levels were below the TK216 concentrations achieved in patients. This heterogeneity has direct implications for clinical translation, suggesting that combination strategies to overcome resistance in the next generation of trials, such as BCL2 or CRM1/XPO1 inhibition, may need to be tailored to the resistance program rather than applied uniformly.

## Author Contributions

**Filippo Spriano:** data curation (equal), formal analysis (equal), investigation (lead), methodology (equal), validation (equal), writing – original draft (equal). **Luciano Cascione:** investigation (supporting), methodology (supporting), writing – review and editing (equal). **Chiara Tarantelli:** investigation (supporting), writing – review and editing (equal). **Giulio Sartori:** investigation (supporting), writing – review and editing (equal). **Alberto J. Arribas:** investigation (supporting), methodology (supporting), writing – review and editing (equal). **Adriana Velasova:** investigation (supporting), writing – review and editing (equal). **Sara Napoli:** methodology (supporting), writing – review and editing (equal). **Ondrej Havranek:** resources (supporting), writing – review and editing (equal). **Jeffrey A. Toretsky:** conceptualization (equal), funding acquisition (equal), writing – review and editing (equal). **Francesco Bertoni:** conceptualization (lead), data curation (equal), formal analysis (equal), funding acquisition (lead), investigation (equal), methodology (equal), project administration (lead), resources (lead), supervision (lead), writing – original draft (equal).

## Funding

This work was supported by Leukemia and Lymphoma Society, #6521‐17. Hyundai Hope on Wheels Professorship in Pediatric Oncology.

## Conflicts of Interest

C.T.: travel grant from iOnctura. L.C.: institutional research funds from Orion; travel grant from HTG. A.J.A.: travel grant from AstraZeneca, consultant for PentixaPharm. O.H.: travel grant from Roche. J.A.T.: co‐inventor on a series of patents held by Georgetown University. F.B.: institutional research funds from BeiGene, Floratek Pharma, Helsinn, HTG Molecular Diagnostics, Ideogen AG, Idorsia Pharmaceuticals Ltd., Immagene, ImmunoGen, iOnctura, KoDiscovery, MabTree, Menarini Ricerche, Oncternal Therapeutics, Spexis AG; consultancy fee from BIMINI Biotech, Helsinn, Menarini; advisory board fees to institution from Novartis; travel grants from Amgen, Astra Zeneca, BeiGene, InnoCare, iOnctura. The other authors declare no conflicts of interest.

## Supporting information


**Figure S1:** Dose–response curve of parental and TK216‐resistant U2932 cells treated with Vorinostat.
**Figure S2:** Vulcano plot of deregulated genes in Cluster A and Cluster B compared to parental cells. (A) Vulcano plot of deregulated genes in Cluster A compared to parental cells. (B) Vulcano plot of deregulated genes in Cluster B compared to parental cells. The top 50 downregulated genes are highlighted in blue, and the top 50 upregulated genes are highlighted in red. Genes ranked based on FC with a minimal −log10 *p* value of 1.3.
**Figure S3:** Gene sets upregulated or downregulated in resistant Cluster A and B (left and right panel, respectively). GSEA, NES, normalized enrichment score obtained with gene‐set enrichment analysis. Red bars = positive NES; Blue bars = negative NES. Upregulated gene‐sets in the resistant Cluster A have positive NES, while downregulated genes have negative NES.
**Figure S4:** Immunoblot for resistant and parental cells with respective quantification. Representative western blot of at least two biological replicates. M1, membrane 1; M2, membrane 2.
**Figure S5:** An enrichment map analysis comparing Cluster A and B resistant clones was performed. An enrichment map analysis was performed with Cystoscape software to compare gene‐set modulation between Cluster A and Cluster B. Every circle represents a gene set. Upregulated (red) or downregulated (blue) gene‐sets in resistant compared to parental cells are shown in the left and right halves of the circle for Cluster B or Cluster A, respectively.
**Figure S6:** Dose response curve of parental cell line treated with TK216 in the presence of resistant cells' conditioned medium.
**Figure S7:** Resistant clones' mutational status. (A) Heatmap showing the mutation frequency in all genes differentially mutated between parental and resistant clones. Pearson's clustering method was used. (B) The heat map shows genes with missense mutations that happen only in exons.
**Figure S8:** Immunoblotting of baseline expression of antiapoptotic proteins in TK216 parental and resistant clones. Representative immunoblot of BCL2, Bcl‐xL, and MCL1 protein expression. Vinculin was used as a loading control. M1, Membrane 1; M2, Membrane 2.

**Table S1:** Transcriptomic signature distinguishing TK216‐resistant cluster A and B versus parental cells. The list shows log2 fold change (resistant vs. parental) and *p* values for all quantified genes. Negative values indicate downregulation in resistance compared to parental cells, and positive values indicate upregulation. A two‐tailed unpaired *T*‐test was used.

**Table S2:** Compound sensitivity profile in TK216‐resistant versus parental cells across a targeted inhibitor library. The table reports percent viability relative to the vehicle for parental and two resistant cell lines representative of cluster A and B (resistant clone 2 for cluster A and resistant clone 3 for cluster B) at two concentrations (50 nM and 500 nM).

## Data Availability

Expression data will be available in NCBI GEO.
